# Structural Change and Dynamics of Pakistan Stock Market during Crisis: A Complex Network Perspective

**DOI:** 10.3390/e21030248

**Published:** 2019-03-05

**Authors:** Bilal Ahmed Memon, Hongxing Yao

**Affiliations:** School of Finance and Economics, Jiangsu University, Zhenjiang 212013, China

**Keywords:** complex network, stock correlation network, Shannon entropy, threshold network, minimum spanning tree, crisis, Pakistan stock exchange

## Abstract

We studied the cross-correlations in the daily closing prices of 181 stocks listed on the Pakistan stock exchange (PSX) covering a time period of 2007–2017 to compute the threshold networks and minimum spanning trees. In addition to the full sample analysis, our study uses three subsamples to examine the structural change and topological evolution before, during, and after the global financial crisis of 2008. We also apply Shannon entropy on the overall sample to measure the volatility of individual stocks. Our results find substantial clustering and a crisis-like less stable overall market structure, given the external and internal events of terrorism, political, financial, and economic crisis for Pakistan. The subsample results further reveal hierarchal scale-free structures and a reconfigured metastable market structure during a postcrisis period. In addition, time varying topological measures confirm the evidence of the presence of several star-like structures, the shrinkage of tree length due to crisis-related shocks, and an expansion in the recovery phase. Finally, changes of the central node of minimum spanning trees (MSTs), the volatile stock recognition using Shannon entropy, and the topology of threshold networks will help local and international investors of Pakistan Stock Exchange limited (PSX) to manage their portfolios or regulators to monitor the important nodes to achieve stability and to predict an upcoming crisis.

## 1. Introduction

Due to globalization and financial integration, stock markets throughout the world are strongly interconnected. For example, the Global financial crisis (hereafter, GFC) that begun from the USA in 2 April 2007 has affected almost all of the financial markets of the world [1]. The propagation of risks and the complex nature of external and internal events to a local stock market require a thorough study of the stock correlation networks and their structural dynamics. Introduced by Mantegna [2], the correlation-based networks are widely used in the financial network literature to quantify the impact of various crisis events [3,4,5,6,7,8,9,10,11]. An extension of the Minimum spanning tree (MST) method for the correlation network was later on presented by Tumminello et al. [12], known as planar maximally filtered graph (PMFG), and Boginski et al. [13] formed a correlation threshold (CT) network. The uncertainty of the stock market and the volatility in stock market returns can be measured with entropy-based approaches, as suggested by previous studies [14,15,16,17,18,19]. Most importantly, a complex system such as the stock market presents its structure better when it is under stress. 

While studying the US stock market, Onnela et al. [20] discover structural changes and a shrinkage in the tree length due to crises by using the correlation network of dynamic asset trees. In addition, Vandewalle et al. [21] and Nobi et al. [22] found a power-law degree distribution of the US stock market. Li et al. [23] show a star-like minimum spanning tree (MST) topology for the Euro Stoxx market during a crisis. Dimitrios and Vasileios [24] highlight the importance of a few stocks that can influence the entire Greek stock market. While examining the South African stock market, Majapa and Gossel [5] found a shrinkage in the tree length during a crisis and a growth afterwards. More interestingly, Kantar et al. [25], after applying MST, showed no impact of the global financial crisis 2008 on Turkish firms. Examining Asian capital markets, Bhattacharjee et al. [26] observed similar hubs and a decrease in the height of clusters during a crisis. Sensoy and Tabak [27] found a deteriorated network stability with the removal of the Hongkong stock market from the Asia Pacific spanning trees network. Using MST and a hierarchical tree, Yang et al. [28] mentioned the core nodes that should be monitored to maintain the stability and a slight increase in the clustering degree during a financial crisis for China’s stock market. Recently, Nie and Song [29] exhibited the integration of entropy and the dimension of financial correlation-based networks among stock markets of three countries: China, the UK, and the US. It is worth noticing that there are a lot of local stock markets that need to be explored via complex network methods, as past research is targeted at a few stock markets of the world.

In this article, we thoroughly analyze the correlation structure network and dynamics of *N* = 181 stocks from 33 sectors listed on the Pakistan stock exchange (PSX) over a wide period from 2007 to 2017. We observe that the Pakistan stock market experiences severe downward fluctuation due to a financial and trade contagion emerging from the GFC. Therefore, our main aim is to investigate the impact of GFC on the network structure of the Pakistan stock market by diving the timeline into three subperiods. The novelty of this research lies in the network analysis of an overall and period-wise comparison of the pre-financial crisis, the financial crisis, and the post-financial crisis of PSX; that, to best of our knowledge, has not been done in the literature. We first measure the individual stock volatility by applying Shannon entropy on all stocks. Thereafter, we construct the Pakistan stock market network using Pearson correlation coefficients and present the topological properties of nine threshold networks around the GFC. In addition, we apply a physics-derived technique of MST to the entire timeline and three targeted subperiods to study the overall and period-wise structures of PSX and to inspect the scale-free properties of four MST networks. Finally, we present time varying topological measures of the Pakistan stock market to inspect the dynamic evolution of the network. 

The paper is organized as follows: Section 2 reviews the relevant prior work on financial market networks. In Section 3, we describe the data and methodology used in this work. Section 4 shows the empirical results and discusses the results. Finally, we conclude the paper in Section 5. 

## 2. Literature Review

For a stock market, the network approach has appeared as a useful measure to analyze its static and dynamic properties [30,31,32,33]. With regards to the application of a network-based approach to examine the developed markets of the world, Bonanno et al. [34] applied an MST and hierarchical tree (HT) to investigate the major 100 stocks listed in the New York stock exchange (NYSE) over the period of 1995 to 1998. Their results showed clusters of stocks in their respective economic sector, and information on the tree topology led to a portfolio optimization. Similarly, Ulusoy et al. [35] used MST and HT on the top 40 companies of UK listed on the London stock exchange between January 2006 and November 2010. In addition to identifying the common clusters, their results also represented an important role of the economic factors influencing a special group of stocks. Onnela et al. [7] investigated the impact of the black Monday crisis on 116 companies of S&P 500 between 1982 and 2000, using the MST methodology. Their results showed a decrease in the normalized tree length and a reconfiguration of the stocks during the crisis time. Brida and Risso [36] analyzed 29 main German companies of the blue-chip DAX 30 index trading on the Frankfurt stock exchange between January 2003 and November 2008. After using MST and HT, their results revealed linkages among companies with the same branch of economy. Additionally, they found a structural break in the expansion of global distance after implementing bootstrap simulations. Lee et al. [37] examined the high-frequency data of 50 stocks listed in the Korean stock market over the period of January 2009 to December 2009. After constructing MST maps, their results found dense structures with a higher market volatility.

Regarding developing countries’ stock markets, Zhang et al. [38] found a power-law degree distribution and a small-world property of a high frequency time series of the Shanghai stock index between 5 March 2007 and 16 March 2007. Huang et al. [39] presented a structural and topological analysis of threshold networks among 1080 stocks listed in the Shanghai and Shenzhen stock markets of China between 2003 and 2007. Their results showed both a topological robustness and a fragility against random node failures. Nguyen et al. [40] examined companies listed on the Hochiminh Stock Exchange (HSX) of Vietnam over the period of 2008 to 2017. Their results showed star-like MST during a Vietnamese financial crisis period in the year 2011–2012. Bahaludin et al. [41] identified four highly dominant stocks of the Malaysian stock market by using the MST method on the top 100 companies from 2011 to 2013. Tabak et al. [42] applied MST on the Brazilian stock market and found a respective importance of various sectors by using the data of 47 stocks between January 2000 and February 2008.

To fix the distortion from correlation coefficients [43], Lyocsa et al. [32] constructed an MST from the dynamic conditional correlations (DCC) of the US stock market over various sample periods. With the exception of the oil and gas industry, their results revealed heterogeneity among various industry sectors. Additionally, they suggested the DCC approach over rolling correlations while describing the limitations of both methods. Examining nonstationary time series, Ferreira et al. [44] applied a detrended cross-correlation analysis (DCCA) method to study the financial integration among 10 Eurozone countries. Their results showed a dissimilar financial integration among a number of EU countries. Furthermore, Peron et al. [45] mentioned entropy-based methods to examine the topology and dynamic evolution of financial market networks, especially during crisis. However, we construct a network based on Pearson correlation coefficients because it is widely applied in the financial network literature. Additionally, a network based on the correlation of stock returns consists of all the information regarding the stock relationship, including investor expectations.

## 3. Data and Methodology

We analyze the daily closing prices for 181 stocks listed in the Pakistan stock market from 3 January 2007 to 29 December 2017, consisting of 2722 trading days. Previous studies mention a varied time period for GFC for Asian countries (see, for example, the Asian market Indices [46], Japan [47], China [48], Korea [22], and Malaysia [49]). However, the Pakistan stock market experienced severe turbulence and country’s benchmark Karachi stock exchange (KSE-100) index declined rapidly from 14,956.82 points on the first trading day of May 2008 to a plunge in the index value by almost 35.29% or by 5278 points within three months, representing a financial crisis hit. Thus, to capture the full essence of a topological evolution of GFC on PSX, we divide the overall time series into three subperiods: precrisis (8 March 2007 to 2 May 2008), crisis (5 May 2008 to 30 June 2009), and postcrisis (1 July 2009 to 19 August 2010); each subperiod comprises 285 trading days. Table 1 mentions 33 sectors under the investigation of the Pakistan stock market network. A complete list of 181 stocks acting as nodes of the PSX network in a chronical order and categorized by their respective industry sectors is mentioned in Appendix A.

A set of n stocks is represented by S={i|i=0,1,…,n}, where the individual stock corresponds to a numerical label i in S. We define $\left\{P_{i}\left(t\right)\right\}$ as the stock i closing price, the log return $r_{i}\left(t\right)$ of stock i after the time interval (Δt) can be calculated as

(1) $$r_{i}\left(t\right)=\ln\left(P_{i}\left(t\right)\right)-\ln\left(P_{i}\left(t-1\right)\right)$$

Since, the volatility of each stock is a latent variable, a proxy needs to be determined. A well-known proxy to examine stock market volatility has been the standard deviation σ. However, we apply the Shannon entropy [50], an alternative way commonly used in the statistical physics of complex dynamics. Given the probability distribution of occurrence $P_{i},\left(i=1,\ldots ,N\right)$, the Shannon entropy $H\left(p_{1},p_{2},\ldots ,p_{n}\right)$, reads

(2) $$H=-\overset{N}{\underset{i=1}{\sum }}p_{i}\log_{2}p_{i}$$

where 0log0 is described as 0 and the normalized related probabilities is $\overset{N}{\underset{i=1}{\sum }}p_{i}=1$. The base 2 for log is drawn so that the computation is given concerning bits of information. We divide the log return $r_{i}\left(t\right)$ of the stock into N different bins and then compute the probabilities of each state i divided by the total number of values of stock S. We then apply the Shannon entropy depending upon the number of selected bins for each stock to measure the uncertainty and volatility (for a detailed study, please see Reference [51]).

Thereafter, we calculate the Pearson correlation coefficient among all pairs of daily returns of stock i and j in set S, given as

(3) $$C_{ij}=\frac{\langle r_{i}r_{j}\rangle -r_{i}\langle r_{j}\rangle }{\sqrt{\left(\langle r_{i}^{2}\rangle -\langle r_{i}\rangle {}_{}^{2}\right)\left(\langle r_{j}^{2}\rangle -\langle r_{j}\rangle {}_{}^{2}\right)}}$$

where $r_{i}$ and $r_{j}$ are the returns of stock i and j and the notation 〈…〉 represents the mean value over the period of investigation. Following this method, we can obtain (181×181) cross-correlation symmetric matrices among all nodes that vary from −1 (negatively correlated) to +1 (positively correlated). We obtain threshold network θ by assigning a certain value to θ, (−1≤θ≤1), from the cross-correlation coefficients. If $C_{ij}$ between two stocks is greater than θ, we build an undirected link between stocks i and j. Perhaps, with same number of nodes for a certain θ, we obtain different set of links [39,52].

In order to construct a minimum spanning tree (MST), we further transform the correlation matrix of (181×181) stocks to a matrix that apprehends the distance in the tree network, as proposed by Mantegna [2] and by Mantegna and Stanley [53]. It is defined as

(4) $$d_{ij}=\sqrt{2\left(1-C_{ij}\right)}$$

The distance $d_{ij}$ among stocks i and j, the MST, denoted as T, is then computed from a data metric of N×(N−1)/2 links to a minimized total weight of V−1 isolated edges, using the Kruskal algorithm [54].

(5) $$T=\underset{\left(i,j\right)\epsilon T}{\sum }d_{ij}$$

## 4. Results and Discussion

In this section, we present findings of the Pakistan stock market correlation network of 181 stocks from 33 industry sectors between January 2007 to December 2017 measured by logarithmic returns.

### 4.1. Correlation Coefficients and Distance Matrices 

**Figure 1:**
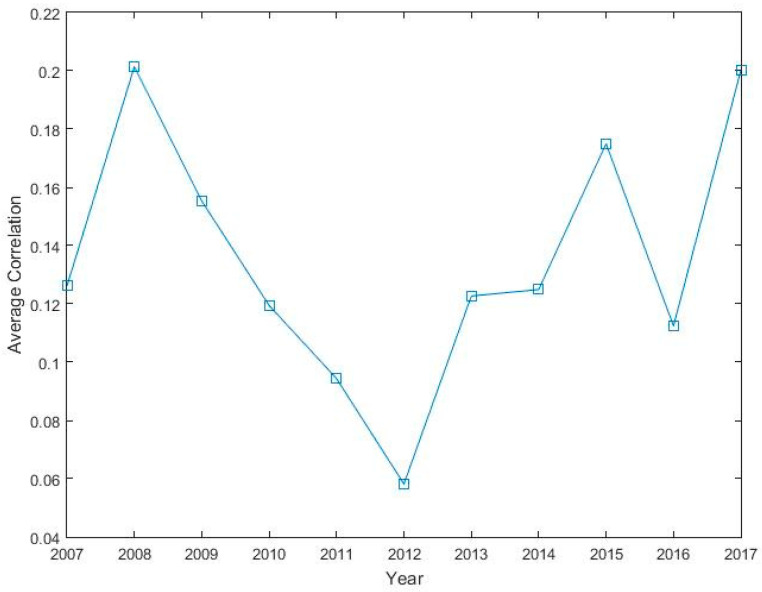
The average cross-correlation coefficients of 181 stocks of the Pakistan stock exchange (PSX).

Figure 1 presents a graph of the average cross-correlation coefficients (CCC) for 181 stocks of the Pakistan stock market between 2007 and 2017. The average CCCs show a tremendous increase in the year 2008 when a GFC struck Pakistan and a decline abruptly after crisis. A local peak in the average CCC can be seen in the year 2017, when country experienced a severe political and economic crisis. The strong correlation among stocks is an indication that common shock was shared by all stocks during crisis period [55]. Pakistan’s economy was sternly hit due to GFC and the country’s GDP growth rate has shown a reduction from 4.833% in the year 2007 to 1.701% in the year 2008. Further, in Table 2, we mention statistics of the Pearson correlation and the distance metrics of the overall and three subperiods around the GFC of the Pakistan stock market. The full sample mean correlation among the stocks of PSX remain at 0.128 and the average distance remains at 1.319, which is marginally lower than the overall sample mean correlation of 0.145 for the South African stock market [5] and, therefore, shows a lower clustering and homogeneity on the Pakistan stock market compared to the South African stock market. In addition, the results reveal a lower mean correlation during the postcrisis period, thus showing comparatively weaker clusters. In contrast, the mean correlation among stocks increases around 39.42% during the crisis period compared to the precrisis period and stabilized to the mean correlation of 0.134 in the postcrisis period, moderately lower than the precrisis mean correlation of 0.137.

### 4.2. Shannon Entropy

**Figure 2:**
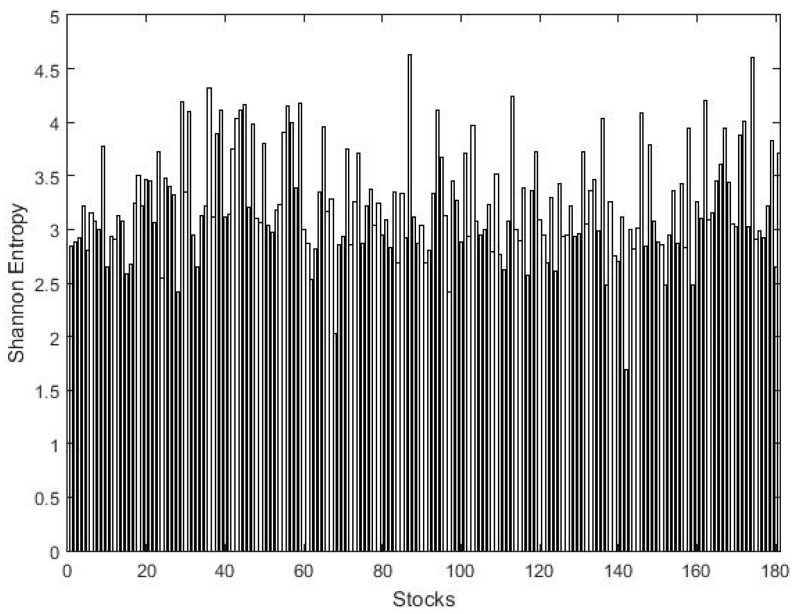
The Shannon entropies of 181 stocks on the PSX with bins of size 0.01.

**Figure 3:**
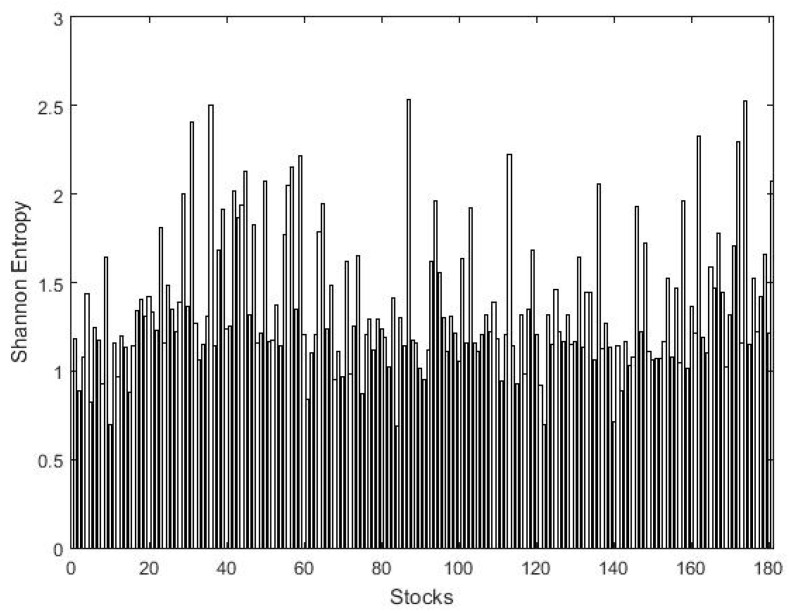
The Shannon entropies of 181 stocks on the PSX with bins of size 0.05.

We calculate the Shannon entropy of N=181 stocks of PSX with two different bin choices of sizes 0.01 and 0.05. Obviously, the result of the first bin size of 0.01 will always be higher than of the other bin size of 0.05 and contains more information than the second bin size [51,56]. The result of the overall sample period is presented in Figure 2 and Figure 3, where a high value of the Shannon entropy represents the most volatile stocks. The results show prominent variation among stocks with a larger bin size; that is why it is preferred in literature. After ranking the entire sample based on the Shannon entropy score, we present the top five most and least volatile stocks of PSX in Table 3. The results show that Invest capital investment bank (ICIBL) carries the highest entropy score of 4.634 with a bin size of 0.01 and, therefore, is the most volatile stock in the PSX. Simultaneously, Pakistan services ltd. (PSEL) is the least volatile stock of PSX with a lowest Shannon entropy score of 1.694 among the entire sample. Furthermore, the average entropy of the investment and securities companies sector remains the highest among the entire sample, 3.923, with a bin size 0.01, followed by the textile weaving sector average entropy of 3.827, representing the most volatile sectors of the PSX.

### 4.3. Threshold Network

In this subsection, we present the topology of correlation threshold networks that have been achieved after analyzing three subperiod metrics (precrisis, crisis, and postcrisis). It means that a line is drawn acting as the undirected link for stocks at three different correlation θ values of $C_{ij}>0.1$, $C_{ij}>0.3$, and $C_{ij}>0.5$ and that nine adjacency matrices are created for three different subperiods. The results in Table 4 exhibit a dense network for all the subperiods at θ>0.1, particularly for the crisis period with a high network density of 0.674 and with 67.37% of the retaining edges in comparison with the other two periods. However, the density of the threshold network reduces significantly at θ>0.5, since a higher threshold value corresponds to fewer edges [57]. The density of the crisis period at θ>0.5 remains high to 0.183 in comparison with the precrisis and postcrisis periods due to a tight correlation among stocks, which is a sign of instability because markets tend to act as one during crises [58]. In addition, a high number of 86 stocks acting as nodes in the threshold network are connected at θ>0.5 for the crisis period in comparison with 37 stocks in the precrisis and 49 stocks in the postcrisis periods. Regarding sectoral influence, the cement sector nodes of Fauji cement company (FCCL) and DG Khan cement company (DGKC) are key nodes in the threshold network during the precrisis period. Whereas, DGKC dominates in the crisis period threshold network by forming a major cluster at a θ value of 0.3 and higher, along with the fertilizer sector important node of Engro corporation (ENGRO). However, the period after crisis presents important nodes with many links from three sectors of investment companies, cement, and fertilizers.

### 4.4. Minimum Spanning Tree

**Figure 4:**
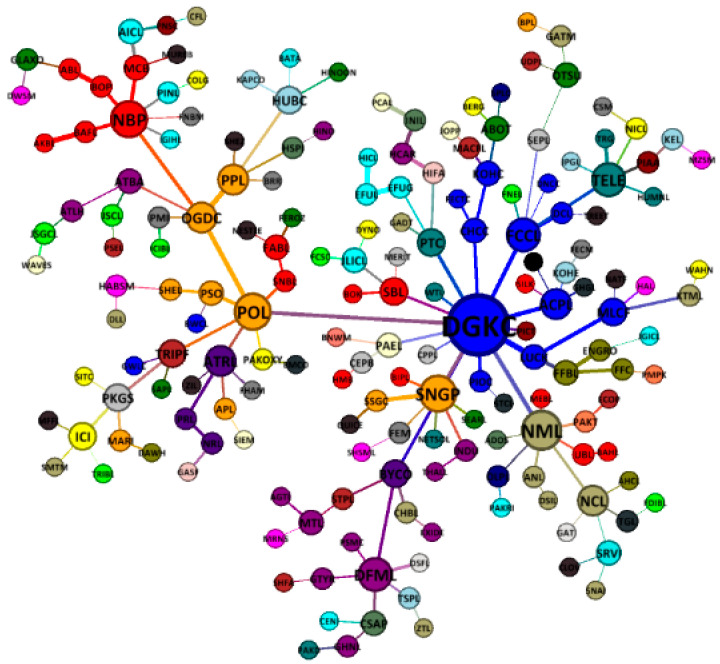
A precrisis minimum spanning tree map of 181 stocks on the PSX network (8 March 2007 to 2 May 2008).

We construct four minimum spanning trees of the Pakistan stock exchange network for three subperiods around a GFC and a full sample period to study the evolving connectivity and efficacy of nodes (all nodes are colored according to their respective sector (please see Appendix A) and are sized based on their centrality score) in the network. The precrisis minimum spanning tree map of PSX is presented in Figure 4. The results show an emergence of three major clusters belonging to the cement sector (blue), the oil and gas sector (orange), and the commercial banks (red). In terms of connectivity (the number alongside each node represents its degree of connections), there is one major hub node of DG Khan cement company (DGKC, 15), along with four minor hub nodes, which are Nishat mills (NML, 8), National bank of Pakistan (NBP, 7), Pakistan oilfields (POL, 7), and Sui northern gas pipelines (SNGP, 7). We can observe the scattered role of commercial bank nodes in the MST such as Soneri bank (SNBL), which is connected to the oil and gas exploration sector node POL; Samba bank (SBL) and SILK Bank (SILK), which are connected to the cement sector key nodes of DGKC and ACPL; United Bank (UBL) and Meezan bank (MEBL), which are connected to the textile composite sector key node of Nishat mills (NML); and so on. This shows that the commercial banks sector plays a lead role in spreading the financial crisis to other sectors in the Pakistan stock market network. 

**Figure 5:**
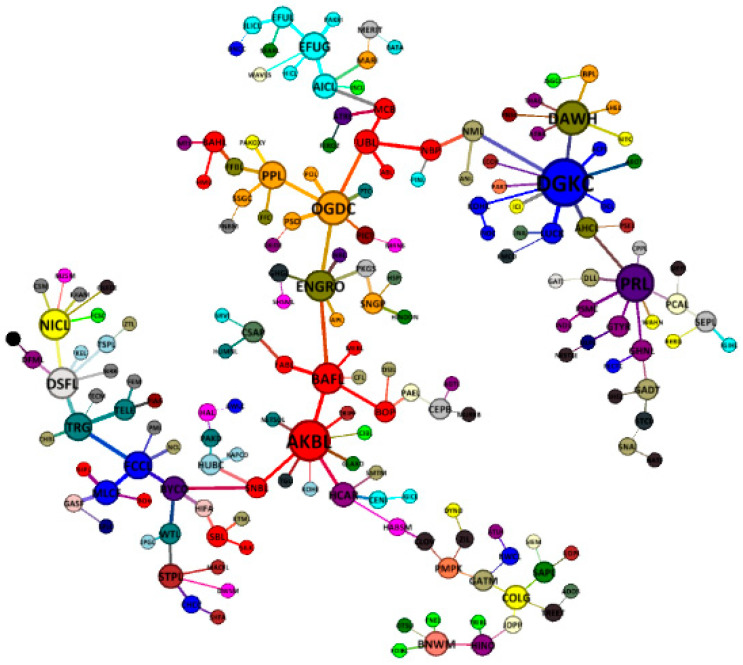
A crisis period minimum spanning tree map of 181 stocks on the PSX network (5 May 2008 to 30 June 2009).

A crisis period minimum spanning tree structure is presented in Figure 5. The results show the appearance of a similar major hub node of DG khan company (DGKC, 11) as in the precrisis period that plays a key role in resisting a crisis shock. Other key nodes with a high degree of connections in the MST are Askari bank (AKBL, 9), Pakistan refinery (PRL, 8), Dawood Hercules Corporation (DAWH, 7), and Oil and gas development company (OGDC, 7). Thus, a crisis MST of PSX reveals a weakening in the number of connections in comparison with the precrisis period, similar to the findings for the South African stock exchange network during crises [5]. In addition, the results also show the importance of the commercial banks sector node of Askari bank (AKBL) that holds the highest betweenness centrality score of 9464 in the crisis period MST of the Pakistan stock market, perhaps reflecting a strong intermediary role. 

**Figure 6:**
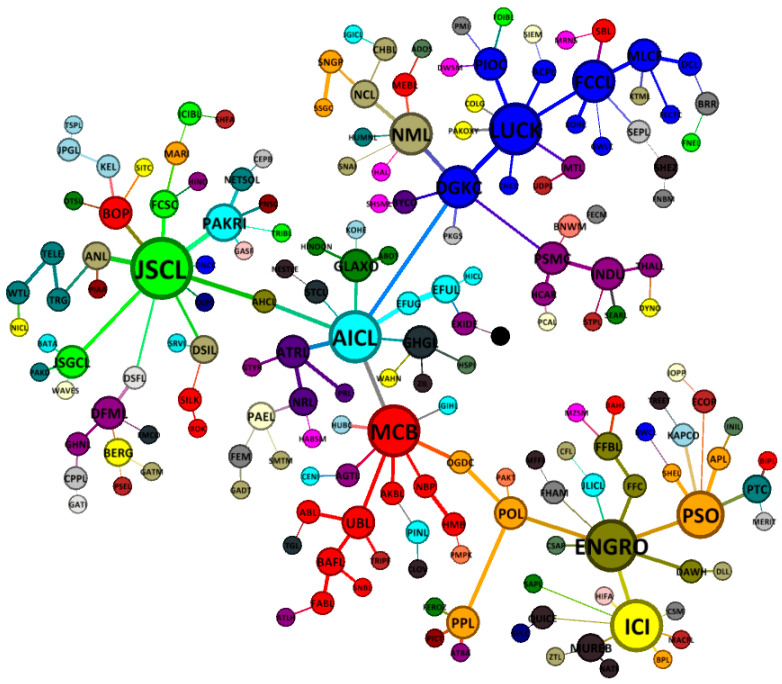
A postcrisis minimum spanning tree map of 181 stocks on the PSX network (1 July 2009 to 19 August 2010).

A postcrisis minimum spanning tree map of PSX network is presented in Figure 6. We can observe that DG khan company (DGKC, 6) is no longer a major hub node as observed in the precrisis and crisis period MST, possibly indicating a changing degree of diversification by the cement sector companies. In addition, there are seven principle nodes in the postcrisis MST, mainly Jahangir Siddiqui company (JSCL, 10), Adamjee insurance company (AICL, 8), ENGRO corporation (ENGRO, 8), ICI Pakistan (ICI, 8), Lucky cement company (LUCK, 8), Muslim commercial bank (MCB, 8), and Pakistan state oil (PSO, 7). The results also show an after-contagion effect in the form of rearrangement and reconfiguration in the MST structure, where commercial banks and cement sector nodes combine themselves among their respective clusters. Thus, a postcrisis MST reduces the impact of connectivity with the riskier sectors of the network. In addition, the results show a compact postcrisis MST structure mainly due to the presence of several hubs that indicate a metastable market structure in comparison with the crisis and precrisis period MSTs [11,59].

**Figure 7:**
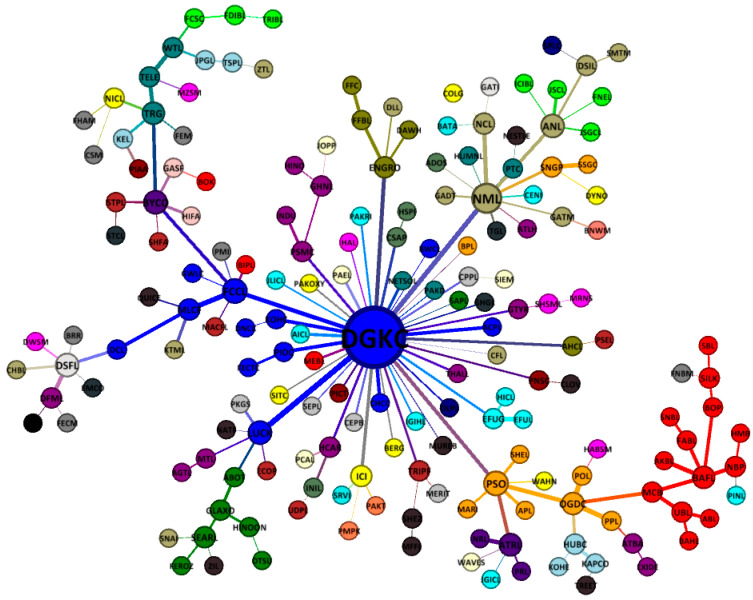
An overall-period star-like minimum spanning tree map of 181 stocks on the PSX (3 January 2007 to 29 December 2017).

Figure 7 presents the overall MST structure of the Pakistan stock market. As can be seen, the whole structure of PSX network revolves around one super hub node of DG khan company having 42 connections, followed by the important nodes of Nishat mills (NML 12), Fauji cement company (FCCL 7), and Pakistan state oil (PSO 7). Hence, the rise and fall of DGKC will give a huge impact on the stability structure of the PSX network, as mentioned by Sharif et al. [60] for the HWAN and MRES nodes of the Malaysian stock market network. The results also reveal a star-like less stable market structure of PSX during the entire period of study, similar to the structures of the Vietnamese stock exchange [40] and German stock exchange [61] during crises. The crisis-like structure is well-suited, given the turbulent timeline of 11 years for Pakistan that posed various challenges and threats, among the major being GFC, terrorism, and economic and political crisis. Furthermore, the results show a substantial clustering on the Pakistan stock exchange network because stocks mostly tend to cluster based on their economic activity.

### 4.5. Scale-Free Strcuture of MSTs

**Figure 8:**
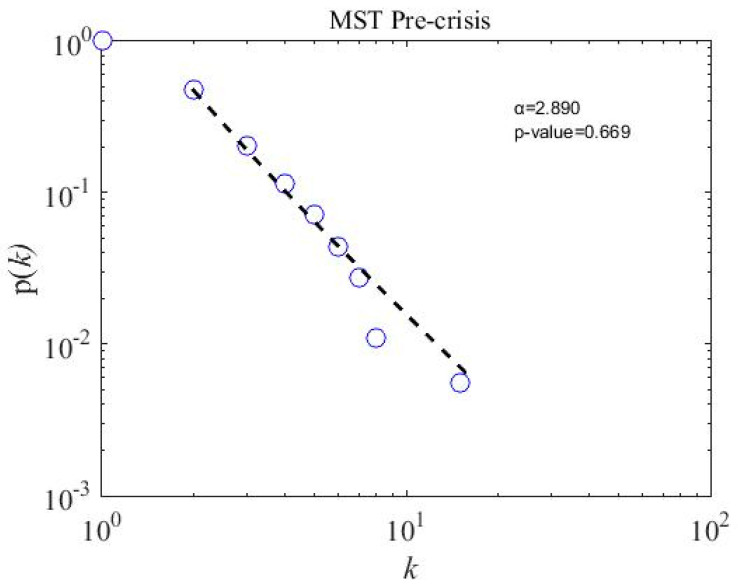
A precrisis minimum spanning tree degree distribution of 181 stocks on the PSX network: the *p*-value is 0.669, which means the stocks follow the power-law distribution.

**Figure 9:**
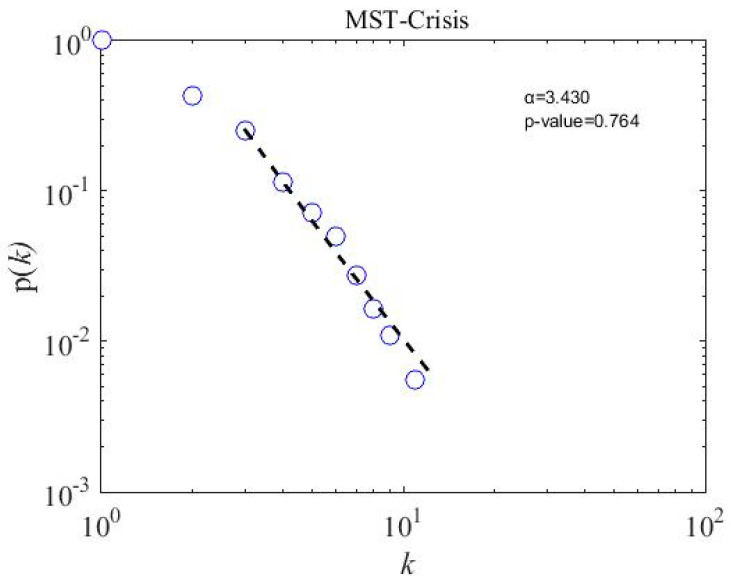
A crisis period minimum spanning tree degree distribution of 181 stocks on the PSX network: the *p*-value is 0.764, which means the stocks follow the power-law distribution.

**Figure 10:**
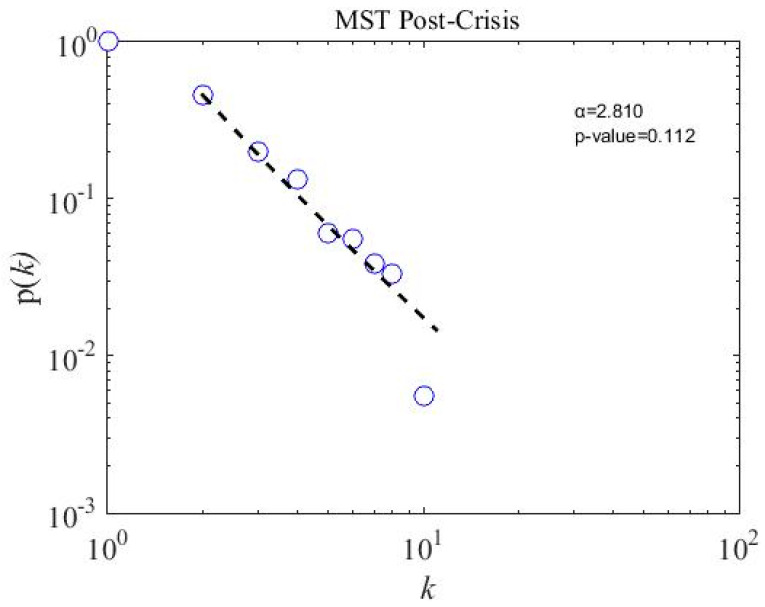
A postcrisis minimum spanning tree degree distribution of 181 stocks on the PSX network: the *p*-value is 0.112, which means the stocks follow the power-law distribution.

**Figure 11:**
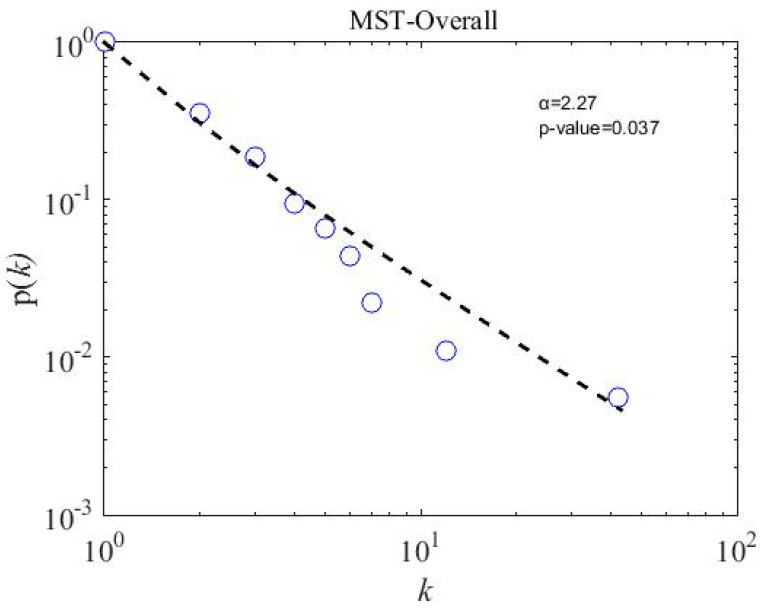
An overall-period minimum spanning tree degree distribution of 181 stock on the PSX.

We calculate the scale-free properties of the MST networks, a concept introduced by Barabasi and Albert in the year 1999 [62] and widely used in financial network literature [20,22,63,64]. The power-law degree distribution p(k) of node i and degree k has a power tail, such as $p\left(k\right)\sim k^{-\alpha }$; the network is said to be scale-free. We apply a powerful tool introduced by Clauset et al. [65] to observe the degree distribution of subsamples and overall MST networks. To accept the power-law hypothesis, the goodness-of-fit p-value must be larger than 0.1 [65]. The fitting results for three subsample periods are presented in Figure 8, Figure 9 and Figure 10. The p-value for three subsamples is larger than 0.1, which means that the degree distribution follows the power law. However, the p-value of the overall sample period stands at 0.037, shown in Figure 11, which implies not to accept the power-law hypothesis. Similarly, a star-like MST is also found by Nguyen et al. [40] for the Vietnamese stock market from the year 2011 to 2012, where the degree distribution does not fit with the power law distribution. In addition, the power-law exponent (the value of the power-law exponent α nearing 1.0 indicates the longer tail distribution) α for the crisis period is 3.430, which is higher than in the precrisis, α=2.890, and postcrisis, α=2.810, periods. Hence, a postcrisis degree distribution of MST has a longer tail distribution in comparison with the precrisis and crisis period MST networks. As can be seen in Figure 8, Figure 9 and Figure 10, the degree distribution of the postcrisis period is more compact than the pre- and crisis period.

### 4.6. Dynamic Structures of MSTs

In order to examine the consistency and dynamic evolution of the Pakistan stock market network, we divide the overall data sample into T=11 rolling windows of width L (where L is the daily returns of N=181 nodes starting from the first trading day of the year in the month of January and ending on the last trading day of the same year in the month of December) [66]. Thereafter, we construct yearly MSTs and present their finding of degree distribution and normalized tree lengths. 

#### 4.6.1. Degree Distribution

**Figure 12:**
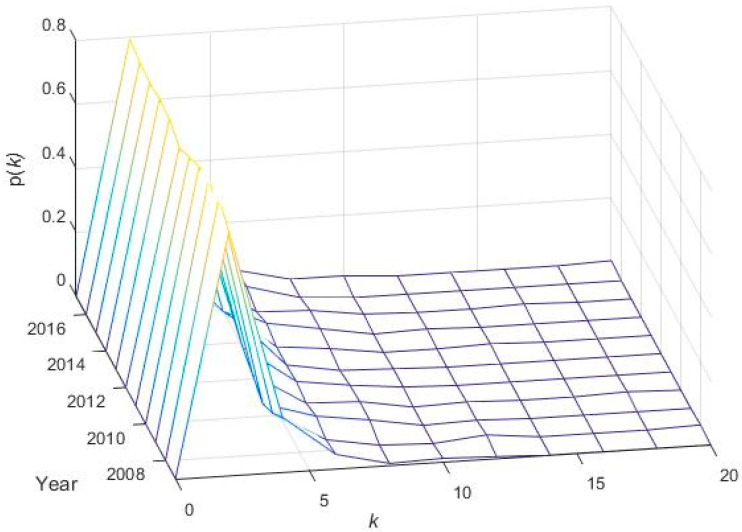
A dynamic minimum spanning tree degree distribution of 181 stocks on the PSX network from January 2007 to December 2017: The x-axis, y-axis, and z-axis mention the degree (k), time (t), and probability p(k), respectively.

The degree distribution p(k) of dynamic MSTs of PSX is presented in Figure 12. We can observe a positively skewed degree distribution representing the heterogeneity of the system. However, the core nodes are largely interconnected in a minor portion, whereas a large number of peripheral nodes contain a relatively low number of linkages. This type of configuration represents several star-like MST structures, especially during the GFC in the year 2008 and the economic and political crisis in the year 2017 for the Pakistan stock market network.

#### 4.6.2. Normalized Tree Length

According to Onnela et al. [67], the normalized tree length (NTL) of MST T=(V,E) can be calculated as follows:

(6) $$L\left(t\right)=\frac{1}{n-1}\underset{\left(i,j\right)\epsilon T}{\sum }d_{ij}\left(t\right)$$

where n is the nodes of the network in T and $d_{ij}$ is the distance among nodes i and j. 

**Figure 13:**
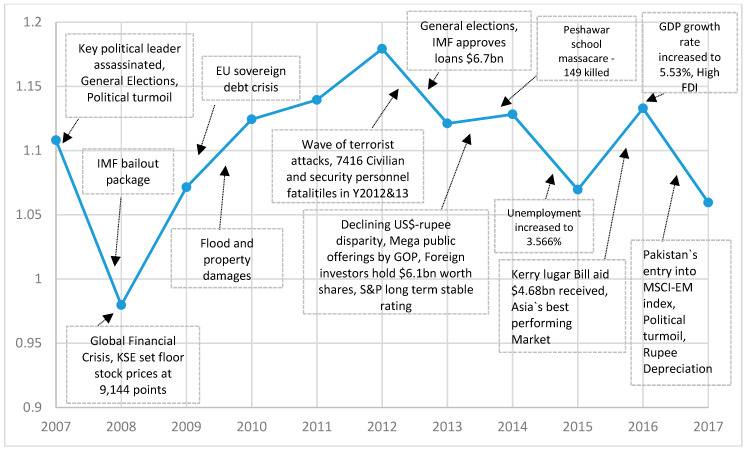
The normalized tree length of a dynamic minimum spanning tree of 181 stocks on the PSX network from January 2007 to December 2017.

Figure 13 shows the time-varying result of a normalized tree length of the Pakistan stock market network. As can be seen, the lowest NTL curve during a GFC is observed for the PSX network in the year 2008 and implies a higher correlation among stocks. However, after getting a financial assistance package from the International monetary fund (IMF) to curb the GFC in the year 2008, the NTL curve shows a gradual increase and recovery that leads to expansion thereafter. In addition, the EU sovereign debt crisis appears to have no significant impact on the PSX network, and so, it is the flood and resultant property damages that affected 14 million people in the year 2010 [68]. To sum up, the results show that the crisis-related shocks of terrorism, politics, and economics resulted in the shrinkage of the PSX network.

## 5. Conclusions

In summary, we have investigated the structural change and dynamic evolution of the Pakistan stock market from January 2007 to December 2017. We applied the Shannon entropy on all 181 stocks acting as nodes in our study to calculate the stock market volatility with two different bins and listed the top five most and least volatile stocks. However, the main aim of our study was to examine the structural change in the Pakistan stock market network around a GFC; therefore, we divided the whole timeline into three different subperiods around a GFC. We show that the correlation among stocks of the Pakistan stock market are at the highest level during the time period of global financial crisis in the year 2008. The subsample results of correlation and distance matrices also reveal a higher mean correlation and resultant lower distances during a crisis period in comparison with the pre- and postcrisis periods. From the topology of nine threshold networks of subperiods, we noticed a comparatively high network density for the crisis period at low thresholds. Similarly, at a larger correlation threshold, a great number of nodes connect with each other during the crisis period, representing a tight correlation and instable market state in comparison with the pre- and postcrisis periods. In addition, we observed scale-free MSTs during the three subperiods and the scattered commercial banking sector in the precrisis, implying that financial crisis spread to other sectors of the Pakistan stock market through the commercial banking sector. The results further showed a metastable market state structure of MST and a recovery in the postcrisis period. Given the turbulent timeline of the overall period of study for Pakistan, the MST of the entire sample period of the Pakistan stock market revealed a crisis-like less stable market structure and the emergence of a super hub node: DG khan cement company (DGKC), belonging to the cement sector. However, a substantial clustering can be seen where nodes connect with each other based on their economic activity. To study the dynamic evolution of PSX, we presented a degree distribution and normalized tree length on 11 year rolling windows that showed several star-like positively skewed networks and a shrinkage of tree lengths due to the crisis-related shocks of terrorism, politics, economics, and finances. 

All of these findings on the structural change and dynamic evolution will assist local and international investors of the Pakistan stock market in successfully managing their portfolios or to regulatory bodies to assess the stock market stability. In the future, we aim to explore the complexity and fractal dimensions of the PSX network.

## Figures and Tables

**Table 1:** The Pakistan stock sectors and their respective color in the minimum spanning tree (MST).

S. No	Sector	Color	Number of Companies
1	Automobile Assembler	Purple	9
2	Automobile Parts and Accessories	Purple	4
3	Cable and Electric Goods	Cream	5
4	Cement	Blue	13
5	Chemical	Yellow	8
6	Close-End Mutual Fund	Rose gold	2
7	Commercial Banks	Red	16
8	Engineering	Hazel Green	4
9	Fertilizer	Olive	5
10	Food and Personal Care Products	Charcoal	9
11	Glass and Ceramics	Gunmetal	4
12	Insurance	Cyan	10
13	Inv. Banks/Inv. Cos./Securities Cos.	Lime	7
14	Jute	Black	1
15	Leasing	Navy	2
16	Leather and Tanneries	Celeste	2
17	Miscellaneous	Brown	7
18	Modarabas	Grey	7
19	Oil and Gas Exploration Companies	Orange	4
20	Oil and Gas Marketing Companies	Orange	6
21	Paper and Board	Silver	5
22	Pharmaceuticals	Green	7
23	Power Generation and Distribution	Light blue	6
24	Refinery	Indigo	4
25	Sugar and Allied Industries	Magenta	6
26	Synthetic and Rayon	Platinum	2
27	Technology and Communication	Teal	7
28	Textile Composite	Khaki	7
29	Textile Spinning	Khaki	4
30	Textile Weaving	Khaki	2
31	Tobacco	Coral	2
32	Transport	Maroon	3
33	Woollen	Salmon	1

**Table 2:** A summary of the observations covering the precrisis, crisis, postcrisis and overall sample period for Pakistan stock exchange (PSX).

	Distance			Pearson Correlation Coefficient		
	Mean	Maximum	Minimum	Mean	Maximum	Minimum
Precrisis	1.311	1.744	0.635	0.137	0.799	−0.521
Crisis	1.265	1.585	0.641	0.191	0.795	−0.255
Postcrisis	1.313	1.554	0.693	0.134	0.760	−0.208
Overall	1.319	1.450	0.786	0.128	0.691	−0.051

**Table 3:** A list of the top five most and least volatile stocks of the Pakistan stock exchange based on the Shannon Entropy results.

Rank	Node	Sector	Entropy with bins 0.01	Entropy with bins 0.05
List of top five stocks with the highest Shannon entropy scores				
1	ICIBL	Inv. Banks/Inv. Cos./Securities Cos.	4.634	2.533
2	TSPL	Power Generation and Distribution	4.607	2.525
3	CSM	Modarabas	4.318	2.503
4	MZSM	Sugar and Allied Industries	4.245	2.226
5	SPLC	Leasing	4.209	2.324
List of top five stocks with the lowest Shannon entropy scores				
1	PSEL	Miscellaneous	1.694	0.887
2	GATI	Synthetic and Rayon	2.025	0.948
3	KAPCO	Power Generation and Distribution	2.415	1.111
4	CFL	Textile Spinning	2.421	1.389
5	SHEZ	Food and Personal Care Products	2.484	1.073

**Table 4:** The topology of the threshold network before, during, and after a financial crisis for PSX.

	Precrisis			Crisis			Postcrisis		
	*p* >0.1	*p* > 0.3	*p* > 0.5	*p* > 0.1	*p* > 0.3	*p* > 0.5	*p* > 0.1	*p* > 0.3	*p* > 0.5
Nodes	181	123	37	181	161	86	181	107	49
Retaining Edges	9684	1250	74	10975	3891	669	9370	1421	94
% of Retaining Edges	59	8	0.45	67.37	23.89	4.11	57.52	8.72	0.58
Average Degree	107.006	20.325	4	121.271	48.335	15.558	103.536	26.561	3.837
Network Diameter	3	5	7	3	9	6	3	5	7
Average Path Length	1.411	2.163	2.545	1.329	2.245	2.399	1.431	1.964	2.777
Graph Density	0.594	0.167	0.111	0.674	0.302	0.183	0.575	0.251	0.08
Communities	5	8	8	4	5	5	5	5	9
Modularity	0.044	0.134	0.459	0.09	0.167	0.273	0.041	0.109	0.417

## Appendix A

**Table A1:** A list of 181 stocks acting as nodes in the network of the Pakistan stock market classified by their respective industry sector and colored accordingly.

Node	Ticker	Company Name	Sector	Color
1	ABL	Allied Bank Limited	Commercial Banks	RED
2	ABOT	Abbot Laboatories (Pakistan) Limited	Pharmaceuticals	GREEN
3	ACPL	Attock Cement (Pakistan) Limited	Cement	BLUE
4	ADOS	Ados Pakistan Limited	Engineering	HAZEL GREEN
5	AGTL	Al-Ghazi Tractors Limited	Automobile Assembler	PURPLE
6	AHCL	Arif Habib Corporation Limited	Fertilizer	OLIVE
7	AICL	Adamjee Insurance Company Limited	Insurance	CYAN
8	AKBL	Askari Bank Limited	Commercial Banks	RED
9	ANL	Azgard Nine Limited	Textile Composite	KHAKI
10	APL	Attock Petroleum Limited	Oil and Gas Marketing Companies	ORANGE
11	ATBA	Atlas Battery Limited	Automobile Parts & Accessories	PURPLE
12	ATLH	Atlas Honda Limited	Automobile Assembler	PURPLE
13	ATRL	Attock Refinery Limited	Refinery	INDIGO
14	BAFL	Bank Al-Falah Limited	Commercial Banks	RED
15	BAHL	Bank Al-Habib Limited	Commercial Banks	RED
16	BATA	Bata Pakistan Limited	Leather and Tanneries	CELESTE
17	BERG	Berger Paints Pakistan Limited	Chemical	YELLOW
18	BIPL	Bankislami Pakistan Limited	Commercial Banks	RED
19	BNWM	Bannu Woollen Mills Limited	Woollen	SALMON
20	BOK	Bank of Khyber Limited	Commercial Banks	RED
21	BOP	Bank of Punjab Limited	Commercial Banks	RED
22	BPL	Burshane LPG (Pakistan) Limited	Oil and Gas Marketing Companies	ORANGE
23	BRR	B.R.R. Guardian Modaraba	Modarabas	GREY
24	BWCL	Bestway Cement Limited	Cement	BLUE
25	BYCO	Byco Petroleum Pakistan Limited	Refinery	INDIGO
26	CENI	Century Insurance Company Limited	Insurance	CYAN
27	CEPB	Century Paper and Board Mills Limited	Paper and Board	SILVER
28	CFL	Crescent Fibres Limited	Textile Spinning	KHAKI
29	CHBL	Chenab Limited	Textile Composite	KHAKI
30	CHCC	Cherat Cement Company Limited	Cement	BLUE
31	CJPL	Crescent Jute Proudcts Limited	Jute	BLACK
32	CLOV	Clover Pakistan Limited	Food and Personal Care Products	CHARCOAL
33	COLG	Colgate Palmolive (Pakistan) Limited	Chemical	YELLOW
34	CPPL	Cherat Packaging Limited.	Paper and Board	SILVER
35	CSAP	Crescent Steel & Allied Products Limited	Engineering	HAZEL GREEN
36	CSM	Crescent Standard Modaraba	Modarabas	GREY
37	DAWH	Dawood Hercules Corporation Limited	Fertilizer	OLIVE
38	DCL	Dewan Cement Limited	Cement	BLUE
39	DFML	Dewan Farooque Motors Limited	Automobile Assembler	PURPLE
40	DGKC	D.G. Khan Cement Company Limited	Cement	BLUE
41	DLL	Dawood Lawrancepur Limited	Textile Composite	KHAKI
42	DNCC	Dandot Cement Company Limited	Cement	BLUE
43	DSFL	Dewan Salman Fibre Limited	Synthetic and Rayon	PLATINUM
44	DSIL	D.S. Industires Limited	Textile Spinning	KHAKI
45	DWSM	Dewan Sugar Mills Limited	Sugar and Allied Industries	MAGENTA
46	DYNO	Dynea Pakistan Limited	Chemical	YELLOW
47	ECOP	Ecopack Limited	Miscellaneous	BROWN
48	EFUG	EFU General Insurance Limited	Insurance	CYAN
49	EFUL	EFU Life Assurance Limited	Insurance	CYAN
50	EMCO	Emco Industries Limited	Glass and Ceramics	GUNMETAL
51	ENGRO	Engro Corporation Limited	Fertilizer	OLIVE
52	EXIDE	Exide Pakistan Limited	Automobile Parts and Accessories	PURPLE
53	FABL	Faysal Bank Limited	Commercial Banks	RED
54	FCCL	Fauji Cement Company Limited	Cement	BLUE
55	FCSC	First Capital Securites Corporation Limited	Inv. Banks/Inv. Cos./Securities Cos.	LIME
56	FDIBL	First Dawood Investment Bank Limited	Inv. Banks/Inv. Cos./Securities Cos.	LIME
57	FECM	First Elite Capital Modaraba	Modarabas	GREY
58	FECTC	Fecto Cement Limited	Cement	BLUE
59	FEM	First Equity Modarba	Modarabas	GREY
60	FEROZ	Ferozsons Laboratories Limited	Pharmaceuticals	GREEN
61	FFBL	Fauji Fertilizer Bin Qasim Limited	Fertilizer	OLIVE
62	FFC	Fauji Fertilizer Company Limited	Fertilizer	OLIVE
63	FHAM	First Habib Modarba Limited	Modarabas	GREY
64	FNBM	First National Bank Modarba	Modarabas	GREY
65	FNEL	First National Equities Limited	Inv. Banks/Inv. Cos./Securities Cos.	LIME
66	GADT	Gadoon Textile Mills Limited	Textile Spinning	KHAKI
67	GASF	Golden Arrow Selected Funds Limited	Close-End Mutual Fund	ROSEGOLD
68	GATI	Gatron Industries Limited	Synthetic and Rayon	PLATINUM
69	GATM	Gul Ahmed Textile Mills Limited	Textile Composite	KHAKI
70	GHGL	Ghani Glass Limited	Glass and Ceramics	GUNMETAL
71	GHNL	Ghandara Nissan Limited	Automobile Assembler	PURPLE
72	GLAXO	GlaxoSmithKline (Pakistan) Limited	Pharmaceuticals	GREEN
73	GTYR	General Tyre and Rubber Co. of Pakistan Limited	Automobile Parts and Accessories	PURPLE
74	GWLC	Gharibwal Cement Limited	Cement	BLUE
75	HABSM	Habib Sugar Mills Limited	Sugar and Allied Industries	MAGENTA
76	HAL	Habib-ADM Limited	Sugar and Allied Industries	MAGENTA
77	HCAR	Honda Atlas Cars (Pakistan) Limited	Automobile Assembler	PURPLE
78	HICL	Habib Insurance Company Limited	Insurance	CYAN
79	HIFA	HBL Investment Fund	Close-End Mutual Fund	ROSEGOLD
80	HINO	HinoPak Motors Limited	Automobile Assembler	PURPLE
81	HINOON	Highnoon Laboratories Limited	Pharmaceuticals	GREEN
82	HMB	Habib Metropolitan Bank Limited	Commercial Banks	RED
83	HSPI	Huffaz Seamless Pipe Industries Limited	Engineering	HAZEL GREEN
84	HUBC	Hub Power Company Limited	Power Generation and Distribution	LIGHTBLUE
85	HUMNL	Hum Network Limited	Technology and Communication	TEAL
86	ICI	I.C.I. Pakistan Limited	Chemical	YELLOW
87	ICIBL	Invest Capital Investment Bank Limited	Inv. Banks/Inv. Cos./Securities Cos.	LIME
88	IGIHL	IGI Holdings Limited	Insurance	CYAN
89	INDU	Indus Motor Company Limited	Automobile Assembler	PURPLE
90	INIL	International Industries Limited	Engineering	HAZEL GREEN
91	JGICL	Jubilee General Insurance Company Limited	Insurance	CYAN
92	JLICL	Jubilee Life Insurance Company Limited	Insurance	CYAN
93	JOPP	Johnson and Phillips (Pakistan) Limited	Cable and Electric Goods	CREAM
94	JPGL	Japan Power Generation Limited	Power Generation and Distribution	LIGHTBLUE
95	JSCL	Jahangir Siddiqui Company Limited	Inv. Banks/Inv. Cos./Securities Cos.	LIME
96	JSGCL	JS Global Capital Limited	Inv. Banks/Inv. Cos./Securities Cos.	LIME
97	KAPCO	Kot Addu Power Company Limited	Power Generation and Distribution	LIGHTBLUE
98	KEL	K-Electric Limited	Power Generation and Distribution	LIGHTBLUE
99	KOHC	Kohat Cement Limited	Cement	BLUE
100	KOHE	Kohinoor Energy Limited	Power Generation and Distribution	LIGHTBLUE
101	KTML	Kohinoor Textile Mills Limited	Textile Composite	KHAKI
102	LUCK	Lucky Cement Limited	Cement	BLUE
103	MACFL	Macpac Films Limited	Miscellaneous	BROWN
104	MARI	Mari Petroleum Company Limited	Oil and Gas Exploration Companies	ORANGE
105	MCB	MCB Bank Limited	Commercial Banks	RED
106	MEBL	Meezan Bank Limited	Commercial Banks	RED
107	MERIT	Merit Packaging Limited	Paper and Board	SILVER
108	MFFL	Mitchells Fruit Farms Limited	Food and Personal Care Products	CHARCOAL
109	MLCF	Maple Leaf Cement Factory Limited	Cement	BLUE
110	MRNS	Mehran Sugar Mills Limited	Sugar and Allied Industries	MAGENTA
111	MTL	Millat Tractors Limited	Automobile Assembler	PURPLE
112	MUREB	Murree Brewery Company Limited	Food and Personal Care Products	CHARCOAL
113	MZSM	Mirza Sugar Mills Limited	Sugar and Allied Industries	MAGENTA
114	NATF	National Foods Limited	Food and Personal Care Products	CHARCOAL
115	NBP	National Bank of Pakistan	Commercial Banks	RED
116	NCL	Nishat Chunian Limited	Textile Composite	KHAKI
117	NESTLE	Nestle Pakistan Limited	Food and Personal Care Products	CHARCOAL
118	NETSOL	NetSol Technologies Limited	Technology and Communication	TEAL
119	NICL	Nimir Industrial Chemicals Limited	Chemical	YELLOW
120	NML	Nishat Mills Limited	Textile Composite	KHAKI
121	NRL	National Refinery Limited	Refinery	INDIGO
122	OGDC	Oil and Gas Development Company Limited	Oil and Gas Exploration Companies	ORANGE
123	OLPL	Orix Leasing Pakistan Limited	Leasing	NAVY
124	OTSU	Otsuka Pakistan Limited	Pharmaceuticals	GREEN
125	PAEL	Pak Elektron Limited	Cable and Electric Goods	CREAM
126	PAKD	Pak Datacom Limited	Technology and Communication	TEAL
127	PAKOXY	Pakistan Oxygen Limited	Chemical	YELLOW
128	PAKRI	Pakistan Reinsurance Company Limited	Insurance	CYAN
129	PAKT	Pakistan Tobacco Company Limited	Tobacco	CORAL
130	PCAL	Pakistan Cables Limited	Cable and Electric Goods	CREAM
131	PIAA	Pakistan International Airlines Corporation	Transport	MAROON
132	PICT	Pakistan International Container Terminal Limited	Transport	MAROON
133	PINL	Premier Insurance Limited	Insurance	CYAN
134	PIOC	Pioneer Cement Limited	Cement	BLUE
135	PKGS	Packages Limited	Paper and Board	SILVER
136	PMI	First Prudential Modarba	Modarabas	GREY
137	PMPK	Philip Morris (Pakistan) Limited	Tobacco	CORAL
138	PNSC	Pakistan National Shipping Corporation Limited	Transport	MAROON
139	POL	Pakistan Oilfields Limited	Oil and Gas Exploration Companies	ORANGE
140	PPL	Pakistan Petroleum Limited	Oil and Gas Exploration Companies	ORANGE
141	PRL	Pakistan Refinery Limited	Refinery	INDIGO
142	PSEL	Pakistan Services Limited	Miscellaneous	BROWN
143	PSMC	Pak Suzuki Motor Company Limited	Automobile Assembler	PURPLE
144	PSO	Pakistan State Oil Company Limited	Oil and Gas Marketing Companies	ORANGE
145	PTC	Pakistan Telecommunication Company Limited	Technology and Communication	TEAL
146	QUICE	Quice Food Limited	Food and Personal Care Products	CHARCOAL
147	SAPL	Sanofi-Aventis Pakistan Limited	Pharmaceuticals	GREEN
148	SBL	Samba Bank Limited	Commercial Banks	RED
149	SEARL	The Searle Company Limited	Pharmaceuticals	GREEN
150	SEPL	Security Paper Limited	Paper and Board	SILVER
151	SHEL	Shell Pakistan Limited	Oil and Gas Marketing Companies	ORANGE
152	SHEZ	Shezan International Limited	Food and Personal Care Products	CHARCOAL
153	SHFA	Shifa International Hospitals Limited	Miscellaneous	BROWN
154	SHSML	Shahmurad Sugar Mills Limited	Sugar and Allied Industries	MAGENTA
155	SIEM	Siemens Pakistan Engineering Co. Limited	Cable and Electric Goods	CREAM
156	SILK	Silkbank Limited	Commercial Banks	RED
157	SITC	Sitara Chemical Industries Limited	Chemical	YELLOW
158	SMTM	Samin Textiles Limited	Textile Weaving	KHAKI
159	SNAI	Sana Industries Limited	Textile Spinning	KHAKI
160	SNBL	Soneri Bank Limited	Commercial Banks	RED
161	SNGP	Sui Northern Gas Pipelines Limited	Oil and Gas Marketing Companies	ORANGE
162	SPLC	Saudi Pak Leasing Company Limited	Leasing	NAVY
163	SRVI	Service Industries Limited	Leather and Tanneries	CELESTE
164	SSGC	Sui Southern Gas Company Limited	Oil and Gas Marketing Companies	ORANGE
165	STCL	Shabbir Tiles and Ceramics Limited	Glass and Ceramics	GUNMETAL
166	STPL	Siddiqsons Tin Plate Limited	Miscellaneous	BROWN
167	TELE	Telecard Limited	Technology and Communication	TEAL
168	TGL	Tariq Glass Industries Limited	Glass and Ceramics	GUNMETAL
169	THALL	Thal Limited	Automobile Parts and Accessories	PURPLE
170	TREET	Treet Corporation Limited	Food and Personal Care Products	CHARCOAL
171	TRG	TRG Pakistan Limited	Technology and Communication	TEAL
172	TRIBL	Trust Investment Bank Limited	Inv. Banks/Inv. Cos./Securities Cos.	LIME
173	TRIPF	Tri-Pack Films Limited	Miscellaneous	BROWN
174	TSPL	Tri-Star Power Limited	Power Generation and Distribution	LIGHTBLUE
175	UBL	United Bank Limited	Commercial Banks	RED
176	UDPL	United Distributors Pakistan Limited	Miscellaneous	BROWN
177	WAHN	Wah Noble Chemicals Limited	Chemical	YELLOW
178	WAVES	Waves Singer Pakistan Limited	Cable and Electric Goods	CREAM
179	WTL	WorldCall Telecom Limited	Technology and Communication	TEAL
180	ZIL	ZIL Limited	Food and Personal Care Products	CHARCOAL
181	ZTL	Zephyr Textile Limited	Textile Weaving	KHAKI
